# Phase I/II study of S-1 combined with cisplatin in patients with advanced gastric cancer

**DOI:** 10.1038/sj.bjc.6601413

**Published:** 2003-12-09

**Authors:** W Koizumi, S Tanabe, K Saigenji, A Ohtsu, N Boku, F Nagashima, K Shirao, Y Matsumura, M Gotoh

**Affiliations:** 1 Department of Gastroenterology, School of Medicine, East Hospital, Kitasato University, Kanagawa, Japan; 2 Department of Gastrointestinal Oncology/Gastroenterology, National Cancer Center Hospital East, Kashiwa, Japan; 3 Department of Gastrointestinal Oncology/Gastroenterology, National Cancer Center Hospital, Tokyo, Japan

**Keywords:** S-1, CDDP, advanced gastric cancer, cinical benefit

## Abstract

A dose-escalation study of cisplatin (CDDP) combined with S-1, a new oral dihydropyrimidine dehydrogenase inhibitory fluoropyrimidine, was performed to determine the maximum-tolerated dose (MTD), recommended dose (RD), dose-limiting toxicities (DLTs), and objective response rate (RR) in advanced gastric cancer (AGC). S-1 was given orally at 40 mg m^−2^ b.i.d. for 21 consecutive days following a 2-week rest. CDDP was planned to be given intravenously on day 8, at a dose of 60, 70, or 80 mg m^−2^ depending on the DLT. Treatment was repeated every 5 weeks, unless disease progression was observed. In the phase I portion, the MTD of CDDP was presumed to be 70 mg m^−2^, because 33.3% of patients (2/6) developed DLTs, mainly neutropenia. Therefore, the RD of CDDP was estimated as 60 mg m^−2^. In the phase II portion, 19 patients including six patients of the RD phase I portion were evaluated. The median administered courses was four (range: 1–8). The incidences of severe (grades 3–4) haematological and nonhaematological toxicities were 15.8 and 26.3%, respectively, but all were manageable. The RR was 74% (14/19, 95% confidence interval: 54.9−90.6%), and the median survival day was 383. This regimen is considered to be active against AGC with acceptable toxicity.

The significant survival benefit of 5-fluorouracil (5-FU)-based chemotherapy for unresectable advanced gastric cancer (AGC) compared with best supportive care is reported (Murad *et al*, 1993; Glimelius *et al*, 1994; Pyrhonen *et al*, 1995). To improve the objective response rate (RR) and survival for AGC, many combination regimens based on 5-FU and its derivatives have been studied clinically. However, the median survival times (MST) with these combination chemotherapies were only 5.7–10.5 months (Wils *et al*, 1991; Kelsen *et al*, 1992; Kim *et al*, 1993; Vanhoefer *et al*, 2000). Although some combination chemotherapies showing superior results in AGC have been reported (Wils *et al*, 1991; Webb *et al*, 1997), there is no regimen accepted worldwide as the standard treatment (Ohtsu *et al*, 2003). Therefore, we need to develop new agents and combination chemotherapy regimens to achieve greater survival benefit in AGC.

As administered 5-FU is rapidly degraded by dihydropyrimidine dehydrogenase (DPD), DPD seems to be a typical prognosis factor against 5-FU-based chemotherapy (Takabayashi *et al*, 2000). Therefore, a new oral drug that inhibits DPD namely, DPD inhibitory fluoropyrimidine (DIF), was invented (Diasio, 1999). S-1 is a new oral DIF, and consists of tegafur (FT), 5-chloro-2,4-dihydroxypyridine (CDHP), and potassium oxonate (Oxo) at a molar ratio of 1 : 0.4 : 1. It achieved high efficacy without increasing gastrointestinal (GI) toxicity, based on biochemical modulation theory (Shirasaka *et al*, 1996).

In two late phase II clinical studies for AGC in Japan, the combined RR of the two studies was 44.6%, with a very low (2.0%) incidence of grade 3 diarrhoea (Sakata *et al*, 1998; Koizumi *et al*, 2000). S-1 was approved in Japan for AGC under an accelerated approval regulation system in 1999, and for head and neck cancer in 2001, and clinical trials against colorectal (Ohtsu *et al*, 2000), breast (Sano *et al*, 2000), and lung cancer (Kawahara *et al*, 2001) are now ongoing and high responses have been reported. The phase II studies of S-1 against gastric (Chollet *et al*, 2003) and colorectal cancer (den Brande *et al*, 2003) in Europe by EORTC-Early Clinical Study Group also revealed high efficacy. Therefore, S-1 can be anticipated to be one of the key drugs for AGC.

Several combination regimens show high RR; however, toxic effects limited the survival benefit (Kelsen *et al*, 1992; Kim *et al*, 1993; Vanhoefer *et al*, 2000; Ohtsu *et al*, 2003). Therefore, new chemotherapy regimens to achieve survival benefit with low toxicities are needed. Combinations of 5-FU and cisplatin (CDDP) were synergistic in preclinical (Scanlon *et al*, 1986; Yamada *et al*, 1990; Shirasaka *et al*, 1993) and clinical studies (Rougier *et al*, 1994) on AGC with acceptable toxicity. Based on these studies, we conducted a phase I/II study of S-1 in combination with CDDP.

## PATIENTS AND METHODS

### Patients

Prior to entry, tumour size was determined by chest or GI X-ray, endoscopic examination of the upper GI tract, computed tomographic (CT) scan of the abdomen, barium enema, and bone scintigram. A complete blood cell count, liver and renal function test, and urinalysis were executed within 7 days before entry.

The eligibility criteria were as follows: aged 20–74 years; histologically proven unresectable locally advanced or metastatic gastric adenocarcinoma; no prior chemotherapy except adjuvant chemotherapy more than 30 days prior to entry; adequate organ function, defined as haemoglobin >8.0 g dl^−1^, leucocyte count >4 000–12 000 mm^−3^, platelet count >100 000 mm^−3^, serum bilirubin level <1.5 mg dl^−1^, serum transaminase (aspartate aminotransferase and alanine aminotransferase) <100 U l^−1^, alkaline phosphatase (ALP) < twice the upper limit of the normal range (ULN) of each hospital, serum creatinine level less than the ULN of each hospital, creatinine clearance >50 ml min^−1^; Eastern Cooperative Oncology performance status (PS) 0–2; expected survival period more than 3 months; and written informed consent from the patients. Patients with symptomatic brain metastases were not eligible.

This study was approved by the ethics committees in each institution.

### Treatment and dose escalation schedule

S-1 was given orally at a dose that did not exceed 40 mg m^−2^ based on the patient's body surface area (BSA): BSA<1.25 m^2^, 40 mg; 1.25−1.5 m^2^, 50 mg, and BSA>1.5 m^2^, 60 mg, for 21 consecutive days (b.i.d.) and CDDP was diluted in 400 ml physiological saline, and administered as a 120-min i.v. infusion on day 8. The starting dose of CDDP was 60 mg m^−2^ (level 1), which was planned to be increased in 10 mg m^−2^ increments to 80 mg m^−2^ unless maximum-tolerated dose (MTD) was achieved. The starting dose of CDDP corresponded to 66.7−85.7% of the recommended dose (RD) for gastric cancer in Japan. No intrapatient dose escalation was allowed. At least three patients were treated at each dose level. If one of three patients at a given dose developed any dose-limiting toxicity (DLT), other three or more patients were to be entered at the same dose. Before proceeding to the next dose level, all previously treated patients had received at least one course.

This treatment course was repeated every 5 weeks with an allowance for a delay in treatment if toxicity was observed.

To avoid CDDP-induced renal damage, patients were hydrated on day 8 with 1500 ml 5% glucose, and furosemide was given 30 min prior to the start of CDDP infusion, and 4000 ml 5% glucose was continued for another 48 h.

The next course was started only for the patient whose organ biological parameter had been maintained as eligibility criteria, except the leucocyte count (>3000 mm^−3^) and no disease progression observed. Prophylactic administration of antiemetic medication (5-HT_3_ antagonist plus corticosteroid) at standard doses was routinely used when CDDP was administered to prevent nausea and vomiting. The treatment was repeated unless disease progression or severe toxicity was observed. S-1 was provided by Taiho Pharmaceutical Co., Ltd (Tokyo, Japan).

### Evaluation

A complete blood cell count, liver and renal function test, and urinalysis were assessed at least once a week during the first course, and every other week afterwards. Before each course, additional examinations were performed to evaluate sites.

The National Cancer Institute common toxicity criteria version 2.0 was applied to evaluate the toxicity of this therapy. DLTs were defined as grade 4 neutropenia lasting more than 3 days, any febrile grade 3 or 4 (severe) haematological toxicity, or grade 3 nonhaematological toxicity (except nausea and vomiting). It was also categorised as DLT when the second course treatment was not resumed within 18 days after the first course. The MTD was defined as the dose at which 33% or more patients experienced DLTs during the first course.

Tumour responses were evaluated according to the classification of the Japanese Research Society for Gastric Cancer based on its volume, which was estimated by X-ray imaging or CT scan (Nishi *et al*, 1995). A complete response (CR) was defined as the disappearance of all evidence of cancer for at least 4 weeks, and a partial response (PR) was defined as less than complete, but more than 50% reduction of tumour volume for at least 4 weeks without any evidence of new lesions or progression, respectively. No change was defined as less than a 50% reduction or less than a 25% increase without any new lesions. Progressive disease (PD) was defined as a more than 25% increase in a solitary lesion or the appearance of new lesions. Tumour responses of the primary site were evaluated by the roentgenographic and endoscopic evaluation criteria proposed by the Japanese Research Society for Gastric Cancer. The survival period was calculated from the start of treatment to death or the latest followed-up day. The time to remission was defined as the period from the start of treatment to the onset of PR. The duration of PR was defined as the period from the onset of PR to the first day when progression was noted. The eligibility and suitability for assessment and the objective response to the treatment were reviewed extramurally.

### Pharmacokinetics

In the phase I portion, a pharmacokinetic (PK) study was conducted for FT, 5-FU, CDHP, and Oxo on days 6 and 8 during the first course to evaluate if any metabolic interactions between the component of S-1 and CDDP were seen in this study. Whole blood samples were taken before and 1, 2, 4, and 8 h after S-1 administration on days 6 and 8 during the first course.

### Statistics

The PK parameters were compared between patients treated with S-1 alone on day 6, and combined with CDDP on day 8 by paired *t*-test.

## RESULTS

Between April 1999 and July 2000, 25 patients were entered from three participating centres. The first 12 patients were entered into the phase I portion and the next 13 patients were entered into the phase II portion to confirm the toxicities and efficacy at the RD. All patients were eligible for toxicity evaluation in any course and objective response evaluations (Table 1

**Table 1:** Patient characteristics

	**Phase I portion**		**Phase II portion**
**CDDP (mg m^−2^)**	**60**	**70**	**60**
**No. of patients**	**6**	**6**	**19**
*Age (years)*			
Median	60.5	63.5	60
Range	39–71	31–72	39–72
<65	4	5	13
⩾65	2	1	6

*Sex*			
Female	1	2	2
Male	5	4	17

*Performance status*			
0	4	5	14
1	2	1	4
2	0	0	1

*Pathology*			
Intestinal	2	2	6
Diffuse	4	4	13
Gastrectomy	0	1	5
Adjuvant chemotherapy	0	0	1

CDDP=cisplatin.

). Six patients had undergone gastrectomy and one had also received adjuvant chemotherapy after gastrectomy. Although all patients had metastatic lesion, one patient whose lymph node metastasis lesion was too small to evaluate was evaluated only for primary gastric lesion. Histological evaluation revealed eight patients to be intestinal type and 17 patients to be diffuse type. A total of 109 courses were given: 14 patients (74%) received four or more courses, and seven patients (37%) received six to eight courses at level 1 (CDDP: 60 mg m^−2^), three patients (50%) received four or more courses, and one patient (17%) received six courses at level 2 (CDDP: 70 mg m^−2^). The median number of courses and duration of therapy per patient was four (range: 1–8). The median number of course per patient was four (range: 1–8) at level 1, and four (range: 1–6) at level 2, respectively. The median duration of therapy per patient was 140 days (range: 21–280) at level 1, and 100 days (range: 18–187) at level 2, respectively. Seven patients were treated with S-1 alone after this combination therapy whose number of course by S-1 alone was four (range: 1–5). Two patients received reduced CDDP during the second course at each level. One patient received both reduced S-1 and CDDP doses at level 2.

The median number of days until the start of the second course after completion of scheduled S-1 in the first course was 14 (range: 7−21 days) among 18 patients who were treated with two courses or more. Seven out of the 18 patients required more than 14 days interval to start the second course.

### Determination of MTD

In the phase I portion at level 1, one patient developed grade 3 neutropenia during the first course and required 20 days to start the second course, but the other two patients in the same cohort showed no DLT. An additional three patients were enrolled for safety evaluation, but overall only one of the total of six patients developed a DLT at 60 mg m^−2^ of CDDP. As dose level 2, two of six patients exhibited DLTs in the first course, one of whom had grade 4 neutropenia, and the other had grade 4 anorexia concomitant with grade 3 leucopenia, colitis, and febrile neutropenia. The frequency of severe haematological toxicities increased according to the increment of the CDDP dose (Table 2a

**Table 2:** Toxicity incidence

**(a)**	**Phase I portion**				**(b)**	**Phase II portion**	
**Course**	**First course**					**All courses**	
**CDDP (mg m^−2^)**	**60**		**70**			**60**	
**No. of patients**	**6**		**6**			**19**	
**Toxicity/grade**	**All events**	**Grade 3/4**	**All events**	**Grade 3/4**		**All events**	**Grade 3/4**
*Haematological*							
Leucopenia	2	0	6	2		15	1
Neutropenia	4	1	6	3		13	3
Anaemia	2	0	4	2		10	3
Thrombocytopenia	1	0	3	1		10	0

*Nonhaematological*							
Anorexia	5	0	6	1		18	5
Nausea	2	0	3	0		13	3
Vomiting	0	0	2	0		7	2
Diarrhoea	0	0	1	0		6	1

Grade is based on the National Cancer Institute common toxicity criteria, version 2.0.

CDDP=cisplatin.

). Based on these results, dose level 2 was declared as the MTD, and level 1 was declared as the RD in the following phase II portion. Thus no case was treated with the originally scheduled 80 mg m^−2^ CDDP. The phase II portion was continued with treatment of 60 mg m^−2^ CDDP on day 8, and 40 mg m^−2^ S-1 from days 1 to 21 every 5 weeks, followed by a 2-week rest.

### Safety

In the phase II portion, the most frequently observed severe (grades 3 and 4) haematological toxicity was neutropenia (three cases, 16%). Frequently observed nonhaematological toxicities (all events) included anorexia (18 cases, 95%), nausea (13 cases, 68%), and vomiting (seven cases, 37%) even though prophylactic antiemetic medications were given after CDDP infusions. In addition, the overall incidence of diarrhoea was 32% (six out of 19); however, grade 3 diarrhoea was observed only in one out of 19 (5.3%), and recovered within 2 days (Table 2b).

The median number of days at which grade 3 neutropenia occurred was 29 days (range: 26–69 days) at level 1, whereas the median neutrophil nadir was on day 25 (range: 21–28 days) at level 2, with no differences between dose levels.

During this study, two patients received granulocyte colony-stimulating factor because of neutropenia. Incidences of the worst grade toxicities in patients treated with the RD were grade 1 (one case, 5.3%), grade 2 (nine cases, 47.4%), grade 3 (six cases, 31.6%), and grade 4 (two cases, 10.5%), respectively. Neither treatment-related death nor delayed severe toxicity was observed.

### Efficacy

A total of 19 patients were evaluated to determine the RR at the RD. Of these, six patients were treated with the RD of 60 mg m^−2^ CDDP in the phase I portion and 13 patients were treated with the same CDDP dose in the phase II portion. The RR at the RD in the phase II portion was 73.7% (14/19, 95% confidence interval (CI): 48.8−90.9%). The RR of all 25 eligible patients was 76% (19/25, 95% CI: 54.9−90.6%); four patients showed stable disease as their best response, two patients had PD. The median time to progression was 179 days (range: 24–384) in the phase II portion (Table 3

**Table 3:** Objective response rate and time to progression

	**N**	**CR**	**PR**	**NC**	**PD**	**Response (%)**	**95% CI**	**TTP (day)**	**Range**
Phase II portion	19	0	14	3	2	73.7	48.8–90.9%	179	24–384
Total	25	0	19	4	2	76.0	54.9–90.6%	162	24–384

CR=complete response; PR=partial response; NC=no change; PD=progressive disease; CI=confidence interval; TTP=time to progression (median).

). The median time to PR and the median overall durations of response in 19 responders were 29 (range: 24−64) and 162 days (range: 63−244), respectively. Two responders treated at level 1 were able to adapt gastrectomy after four courses of this combination therapy.

Subgroup analysis by tumour lesion and pathological type for the 25 patients showed that the RR was 67% (4/6) for liver metastasis, 76% (16/21) for lymph node metastasis, and 74% (14/19) for primary lesions, and the RR according to pathological type was 75% (6/8) for the intestinal type, and 76% (13/17) for the diffuse type.

The MST of all eligible patients was 383 days (95% CI: 256−569) and 1- and 2-year survival rates were 52 and 20%, respectively. The median follow-up time for survival analysis was 789 days.

### Pharmacokinetics

Plasma PK analysis was performed on samples obtained from 12 patients during the first course of the phase I portion for S-1 components and total platinum (Table 4

**Table 4:** Pharmacokinetic parameters of S-1 component after oral administration of S-1 alone, or with CDDP

	**CDDP: 60 mg m^−2^**		**CDDP:70 mg m^−2^**	
	**AUC_(0–8)_ (ng h ml^−1^)**	***C*_max_ (ng ml^−1^)**	**AUC_(0–8)_ (ng h ml^−1^)**	***C*_max_ (ng ml^−1^)**
*Day 6 (S-1 alone)*				
FT	22724±10693	3517±1392	23860±12059	3378±1574
5-FU	670.9±155.8	136.8±40.3	860.6±466.4	166.3±78.2
CDHP	1193.6±258.1	308.9±95.1	1031.9±125.2	219.7±17.2
Oxo	373.0±196.1	89.9±62.4	291.1±112.9	61.3±26.2

*Day 8 (with CDDP)*				
FT	20930±8631	3236±1119	21192±11401	3104±1572
5-FU	573.8±148.7	111.9±33.6	782.0±326.6	144.9±42.6
CDHP	1054.9±144.8	241.6±62.6	1127.0±191.4	258.2±66.5
Oxo	282.8±99.1	63.2±25.7	335.1±177.2	68.1±35.4

Values are mean+s.d. (*n*=6).

CDDP=cisplatin; FT=tegafur; 5-FU=5-fluorouracil; CDHP=5-chloro-2, 4-dihydroxypyridine; oxo=potassium oxonate.

). There were no significant differences between the two PK parameters of S-1 components on days 6 and 8.

## DISCUSSION

Two phase II studies of S-1 for AGC patients who had not previously received chemotherapy as a single agent were conducted in Japan (Sakata *et al*, 1998; Koizumi *et al*, 2000). Examining those pooled results, the RR rate was 44.6% (45/101, 95% CI: 35.2−54.3), MST was 244 days (95% CI: 172−319), and 1- and 2-year survival rates were 36.6 and 16.5%, respectively. Subgroup analysis of the objective RR in the two S-1 phase II studies revealed that the well-differentiated cell type (intestinal type) was less sensitive to S-1 (RR=35%, 16/46) than the poorly differentiated type (diffuse type, RR=53%, 29/55). The results of phase II studies suggested that S-1 is one of the most active antitumour single agents for AGC patients and is even comparable to recent combination therapies. Based on these data, to achieve more survival benefit, we therefore planned combination therapy of S-1 with another antitumour agent with a different mechanism of action, because this might obtain better efficacy including longer survival, as a clinical benefit.

We selected CDDP as the combination agent to be used with S-1, because CDDP has been widely used in combination therapy for AGC patients (Rougier *et al*, 1994), and synergistic activity with 5-FU and its derivatives has been reported in animal models (Scanlon *et al*, 1986; Yamada *et al*, 1990; Shirasaka *et al*, 1993).

As most toxicities of S-1 in phase II studies appeared at 4 weeks of consecutive S-1 administration, a new combination therapy of S-1 with CDDP was planned in which S-1 was to be administered daily for 3 consecutive weeks, that is, 1 week less than the period at which toxicities such as leucopenia appeared. In addition, CDDP showed the best activity when given 8 days after the start of daily UFT administration (Ichinose *et al*, 1995). Therefore, CDDP was administered on day 8 of 21-day consecutive S-1 administration. In this combination phase I/II study, S-1 was administrated at the RD (80 mg m^−2^ in a day) and the CDDP dose was escalated from 60 mg m^−2^ as level 1 to 70 mg m^−2^ at level 2. According to the results of the phase I portion, the RD of CDDP combined with S-1 was designated as 60 mg m^−2^ with a DLT of myelosuppression, and in the phase II trial S-1 was orally administered daily for 21 consecutive days followed by a 2-week rest, and CDDP (level 1) was intravenously administered on day 8 of every 5-week period. The severity of neutropenia increased with the dose of CDDP in this study, and grade 4 neutropenia was seen in one of six patients (16.7%) when 70 mg m^−2^ of CDDP was administered with S-1. Furthermore, thrombocytopenia became slightly more marked, although it had been infrequent in S-1 single therapy. These results may lead to the conclusion that CDDP dose-dependently increases the myelosuppressive toxicity of S-1.

For the nonhaematological toxicity, GI toxicity, that is, diarrhoea was observed in this combination therapy. The incidence of diarrhoea was 9.9% (total) and 2.0% (grades 3 and 4) by S-1 single therapy (Sakata *et al*, 1998; Koizumi *et al*, 2000). It suggests that in combination with CDDP, the total incidence of diarrhoea was increased a little; however, severe diarhoea was nearly the same and uncommon, and in any study diarrhoea was manageable, similar to those reported for UFT (Ichinose *et al*, 1995; Borner *et al*, 2002).

The PK results of drugs were similar to the previous results obtained from single-agent therapy (Hirata *et al*, 1999). As there was no PK difference for any S-1 component on day 6, before CDDP administration, and on day 8 after CDDP administration, no PK interaction of CDDP in S-1 metabolism was suggested.

In this study, the overall RR of all eligible patients was 76%. The RR in this combination therapy was high, not only in the diffuse type subgroup, 76%, but also in the intestinal-type subgroup, 75%. The MST (383 days) of our study was longer than in the S-1 single-agent phase II study, or other combination chemotherapy phase II results for AGC patients (Sakata *et al*, 1998; Koizumi *et al*, 2000; Kulke, 2000). Based on these data, combination chemotherapy using S-1 and CDDP was suggested to be worthwhile.

It is reported that the vascular endothelial growth factor (VEGF) is more frequently expressed in well-differentiated tumours, and that it promotes angiogenesis in human gastric carcinoma (Tanigawa *et al*, 1997) and also that the prognosis in the group with high VEGF-C expression was significantly poorer than that in the group with lower VEGF-C expression (Takahashi *et al*, 2002). The combination of CDDP with S-1 therapy is reported to show higher response than S-1 alone, in VEGF-positive gastric cancer patients, which may also support the high objective RR and long survival in this phase I/II combination study (Hironaka *et al*, 2002). Due to its potent angiogenic activity, VEGF is supposed to contribute to metastasis including peritoneal metastasis, a representative life-threatening condition in gastric cancer. S-1 showed superior therapeutic efficacy against peritoneal metastasis in nude mice (Mori *et al*, 2003) and clinically, alone (Iwazawa *et al*, 2002) or in combination with CDDP (Nakamura *et al*, 2002). The results suggest good therapeutic effect of S-1 in terms of survival benefit and increased QOL, as a result of improving several symptoms related to peritoneal metastasis.

The effect of S-1 in combination with CDDP in the present study was shown to be as good as in previous studies based on 5-FU (Kulke, 2000), with no increasing toxicity. Even though the present study is only a limited experience, a further phase III study to confirm the efficacy of combination therapy of S-1 with CDDP is warranted.

Oral chemotherapy has the advantage of greater patient convenience and acceptance with potential cost saving. It is reported that if equivalent response is achieved, patients prefer oral to intravenous medication (Liu *et al*, 1997). For fluoropyrimidine, most patients selected oral UFT, which is a kind of DIF rather than intravenous 5-FU (Borner *et al*, 2002), and UFT has been proposed to raplace i.v. 5-FU as a first-line therapy for metastatic colorectal cancer (Stabuc, 2003). The same appears to be true for not only oral S-1 alone but also in combination with CDDP therapy, which needs limited hospitalisation.

In Japan, several phase II studies using 5-FU combined with CDDP have been tested for AGC, employing various dosage and treatment schedules. However, based on the results of the JCOG9205 phase III study, 5-FU single therapy is still recognised as the standard first-line chemotherapy (Ohtsu *et al*, 2003). In addition, currently, a randomised phase III study (5-FU *vs* S-1 *vs* CPT-11 with CDDP) for AGC patients as the first-line chemotherapy is underway in Japan (JCOG9912). We also initiated a randomised phase III study comparing S-1 alone, and with CDDP for AGC. From these two phase III studies, we may be able to evaluate the clinical benefit of the regimen using S-1 in terms of survival benefits and improving the QOL for AGC patients.
